# Intraoperative Periprosthetic Fractures in Total Hip Arthroplasty in Patients With Sickle Cell Disease at King Fahad Hospital Hofuf: A Cross-Sectional Study

**DOI:** 10.7759/cureus.11390

**Published:** 2020-11-09

**Authors:** Mohammad K Alsaleem, Hassan A Alalwan, Abdullah M Alkhars, Abdullah H Al Huwaiyshil, Wejdan M Alamri

**Affiliations:** 1 Orthopedics, King Fahad Hospital Hofuf, Al-Ahsa, SAU; 2 Orthopedics, King Faisal University, Al-Ahsa, SAU

**Keywords:** sickle cell disease: scd, total hip arthroplasty: tha, avascular necrosis: avn, american society of anesthesiologist: asa, hemoglobin: hb

## Abstract

Background

Patients with avascular necrosis related to sickle cell disease (SCD) can be severely disabled by the severe degenerative changes of their hip. Total hip arthroplasty (THA) remains the only surgical option for some of these patients. Total hip arthroplasty can be a challenging procedure, and SCD patients demonstrate high percentages of medical, intraoperative, and postoperative complications and implant failure. Furthermore, the need for THA following avascular necrosis in the Eastern Province of Saudi Arabia is high, and the subsequent risk of periprosthetic fracture is prevalent. Therefore, it is crucial to conduct such a study.

Aim of the study

This cross-sectional retrospective study aimed to assess the prevalence and associated risk factors for periprosthetic fractures during total hip arthroplasty in sickle cell disease patients at King Fahad Hospital Hofuf, Saudi Arabia.

Methods

We collected the data of all SCD patients who had undergone THA during the study period, January 2015 to September 2020. Forty-nine SCD patients who had undergone THA during the study period were included. Patients who had undergone hip hemiarthroplasty, postoperative fractures, or had an indication of THA other than avascular necrosis were excluded. Surgeon factors, assistant factors, and surgical technique were also excluded. We then analyzed the data according to gender, age, BMI, American Society of Anesthesiologists classification, implant fixation type, avascular necrosis stage, proximal femoral morphology, Vancouver classification type, sickle cell type, preoperative hemoglobin (Hb) level, and the risk of periprosthetic fractures. Descriptive statistics were presented using frequency and percentages for categorical variables, and continuous variables were summarized using means ± standard deviations. Independent t-tests and chi-square tests were used to test for associations between categorical variables. At 0.05, the significance level was set.

Results

Of the patients, 32.7% were male and 67.3% were female. 32.7% of the patients had advanced degenerative changes due to avascular necrosis. Among the patients, 20.4% had an intraoperative periprosthetic femoral fracture, 90% had a Vancouver classification class A, and 10% had a Vancouver classification class B1. According to Dorr classification, 75.5% were classified as Dorr A and 24.5% as Dorr B. Of the patients, 48 had an uncemented implant, and only 1 had cemented. The mean perioperative Hb was 9.02 + 2.02, with a minimum of 6 and a maximum of 14. No significant associations were found between the incidence of intraoperative femoral fracture and the demographic variables and the operative profile characteristics. However, a significantly higher rate of fracture was observed in patients operated on the right side compared to patients operated on the left side.

Conclusion

The prevalence of periprosthetic intraoperative fracture among SCD patients at King Fahad Hospital Hofuf was 20.4% during the study period. Even with adequate perioperative management, orthopedic surgeons must be prepared to deal with high rates of intraoperative fracture. No significant association was found between the incidence of intraoperative femoral fracture in SCD patients and the demographic variables and the operative profiles. However, a significantly higher rate of fracture was observed in patients operated on the right side compared to patients operated on the left side.

## Introduction

Total hip arthroplasty (THA) is a saving procedure used to relieve pain and functional disability associated with many hip problems, most commonly, primary hip osteoarthritis, posttraumatic arthritis, and avascular necrosis in sickle cell disease patients [1-6]. Sickle cell disease is endemic in Saudi Arabia due to the high percentage (57.7%) of ﻿consanguineous marriages, rising to more than 80% in some rural areas [7,8]. Sickle cell disease is most commonly found in the Eastern Province of Saudi Arabia, with a prevalence of ﻿145 cases/10,000 population, which increases the risk of avascular necrosis in these patients [7,8].

Avascular necrosis, a chronic condition that can lead to deterioration of the hip joint and degradation of bone tissue due to a lack of blood supply [9]. The Steinberg classification system includes seven stages, from 0 to VI, according to the extent of involvement and disease progression [9]. At stage III and more advanced stages, THA is required [9].

One of the main complications of THA for sickle cell disease (SCD) patients is periprosthetic hip fracture [1-6]. The incidence of fracture is affected by gender, age, race, BMI, implant fixation type, proximal femoral morphology, sickle cell genotype, and hemoglobin (Hb) level [7-12]. The Vancouver classification system classifies periprosthetic hip fracture according to the location, condition of the femoral implant, and value of surrounding femoral bone stock [13].

A systemic review and meta-analysis undertaken in 2014 [14] revealed a high incidence of periprosthetic fractures after THA in females older than 80 years. Some studies have found that the incidence of periprosthetic fracture is higher in whites with low BMIs [1-5]. Proximal femoral morphology can be radiologically classified before reconstruction according to the Dorr classification [15,16]. Dorr type C is more commonly associated with periprosthetic hip fracture than types A and B [15,16]. In addition, many studies have found that uncemented implant fixation is associated with more pain and risk of fracture compared to cemented implant fixation [17,18]. Furthermore, a 2019 meta-analysis [19] revealed a low incidence of periprosthetic fracture in patients treated with cemented implant fixation. A history of SCD and an Hb level <6 g/dl are associated with a greater risk of periprosthetic fracture [11].

In the Eastern Province, particularly in Al Hasa city, many sickle cell patients require THA following avascular necrosis, and the subsequent risk of periprosthetic fracture is high [7,8]. Moreover, periprosthetic fracture largely affects the prognosis of patients who have undergone THA and subsequently increases hospitalization rates threefold [1-6], which can lead to further complications and may result in death.

Therefore, this study was conducted at King Fahad Hospital Hofuf to assess the prevalence and associated risk factors of developing periprosthetic hip fracture in sickle cell disease patients.

## Materials and methods

Study area, sampling, and data collection

A cross-sectional retrospective study was conducted to examine whether gender, age, BMI, American Society of Anesthesiologists (ASA) classification, implant fixation type, avascular necrosis stage, proximal femoral morphology, Vancouver classification type, sickle cell type, and preoperative Hb level were associated with a greater risk of periprosthetic fractures in sickle cell patients treated at King Fahad Hospital Hofuf with THA during the study period, January 2015 to September 2020.

The examined factors were designed and developed through a literature review of periprosthetic intraoperative fractures, and validity was obtained through a review process with experts in the field. Then, the data were collected from the King Fahad Hospital Hofuf database by two orthopedic residents, who had received prior training for this task after obtaining ethical approval.

Forty-nine SCD patients who had undergone THA during the study period were included. Patients who had undergone hip hemiarthroplasty, postoperative fractures, or had an indication of THA other than avascular necrosis (i.e., osteoarthritis, rheumatoid arthritis, traumatic fracture. etc.) were excluded. Surgeon factors, assistant factors, and surgical technique were also excluded.

Study comparison and intervention

We aimed to determine if gender and age were associated with greater risk. The patients’ ages were grouped as follows: young: less than 18 years old; adult: 18-65; and elderly: 65+. To determine if BMI was associated with greater risk, we used the following BMI categories: underweight = <18.5, healthy weight range = 18.5-24.9, overweight = 25-29.9, class 1 obesity = 30 to <35, class 2 obesity = 35 to <40, and class 3 obesity = ≥40. To determines which ASA classification had more risk, we used the flowing categories: ASA 1: a normal healthy patient, ASA 2: a patient with mild systemic disease, ASA 3: a patient with a serious, non-life-threatening systemic disease, ASA 4: a patient with a serious, life-threatening systemic disease, ASA 5: a morbid patient not expected to survive without surgery, and ASA 6: a brain-dead patient [20]. The preoperative Hb levels were classified into 6-9 g/dl, 10-12 g/dl, and >13 g/dl.

Implant fixation was radiologically categorized as cemented or uncemented. Avascular necrosis was radiologically classified by the Steinberg classification system into seven stages: (stage 0) normal or non-diagnostic radiographs, MRI, and bone scan of the at-risk hip; (stage I) normal radiograph, abnormal bone scan and/or MRI; (stage II) cystic and sclerotic radiographic changes; (stage III) subchondral lucency or crescent sign; (stage IV) femoral head flattening; (stage V) narrowing of joint space with or without acetabular involvement; and (stage VI) advanced degenerative changes [21].

Proximal femoral morphology was radiologically classified according to the Dorr classification: (type A) exhibited thick cortices that begin at the distal end of the lesser trochanter and thicken quickly, which lead to the development of a funnel shape and a narrow diaphysal canal; (type B) proximal bone loss and widening of the diaphysal canal; and (type C) substantial loss of cortical thickness resulting in a much wider intramedullary canal and blurred appearance of the cortices of the bone [22].

Fractures of the femur adjacent to the femoral component of a THA were radiographically described using the Vancouver classification system: Type A fractures occur in the trochanteric region and involve either the greater trochanter or the lesser trochanter, and Type B fractures occur around or just distal to the stem of the femoral component, and on the basis of the implant's stability and the surrounding bone stock, they are subclassified [23]. Fractures of type B1 occur around a stable implant. Fractures of type B2 happen around a loose implant with sufficient bone stock. Fractures of type B3 happen around a loose implant with low bone stock. Fractures of Type C occur well distal to a stable femoral element [23].

Statistical analysis

After data collecting using Microsoft Excel (Microsoft Corporation, Redmond, USA), the data were exported to Statistical Packages for Social Sciences, Version 23 (IBM Corp, Armonk, USA), for analysis. Descriptive statistics were presented using frequency and percentages for categorical variables, and continuous variables were summarized using means ± standard deviations. The association of periprosthetic fractures with gender, age, BMI, ASA classification, implant fixation type, avascular necrosis stage, proximal femoral morphology, Vancouver classification type, sickle cell type, and preoperative Hb level was tested using an independent t-test. A chi-square test was used to test for associations between categorical variables. The level of significance was set at 0.05.

## Results

A total of 49 sickle cell patients who had undergone THA during the study period were included. In Table 1, the demographic profile of patients is provided. Of the patients, 16 (32.7%) were male and 33 (67.3%) were female. For the category of BMI, 3 (6.1%) of the patients were underweight, 26 (43.1%) had a normal BMI, 13 (26.5%) were overweight, and 7 (14.3%) had class 1 obesity. The mean age of the patients was 43.43 + 11.69 years; the minimum age was 23, and the maximum age was 68.

**Table 1:** Demographic profile of the patients (n = 49)

Demographic Characteristics		n	%
Gender			
Male		16	32.70
Female		33	67.30
BMI			
Underweight		3	6.10
Normal BMI		26	53.10
Overweight		13	26.50
Class 1 Obesity		7	14.30
Age (mean, SD)			
Mean		41.96	
Standard Deviation		11.42	
Minimum		23	
Maximum		68	

Table 2 shows the operation profile of the patients. The ASA classification for the patients was as following, 3 (6.1%) were categorized into class 1, 28 (57.1%) were categorized into class 2 and 18 (36.7%) were categorized as class 3. As for the operated side, 20 (40.8%) of the patients had the operation on their right side and 29 (59.2%) had the operation in the left side. The mean perioperative hemoglobin for the patients was 9.02 + 2.02. The minimum hemoglobin was 6 and the maximum hemoglobin was 14.

**Table 2:** Operative profiles of sickle cell disease patients who underwent total hip arthroplasty (n = 49)

Demographic Characteristics		n	%
American Society of Anesthesiologist Class			
1		3	6.10
2		28	57.10
3		18	36.70
Operated Side			
Right		20	40.80
Left		29	59.20
Implant Type Used			
Cemented		1	2.00
Uncemented		48	98.00
Perioperative Hemoglobin			
Mean		9.02	
Standard Deviation		2.02	
Minimum		6	
Maximum		14	

**Figure 1:**
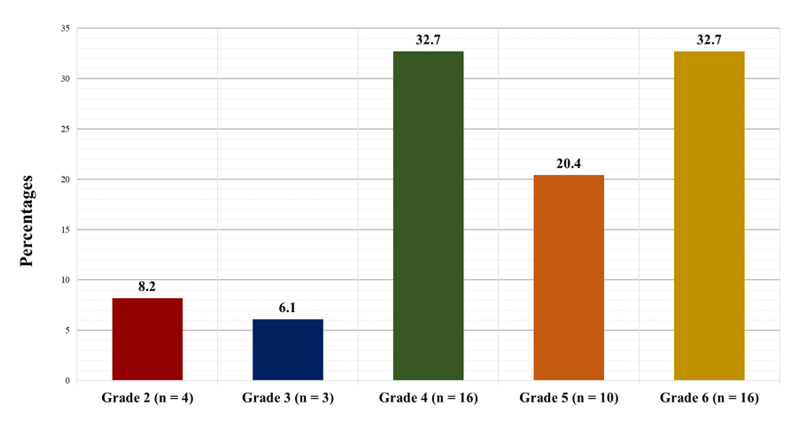
Avascular necrosis staging using the Steinberg classification system

Figure 1 displays the avascular necrosis staging of the SCD patients who underwent THA using the Steinberg classification system. Four patients (8.2%) had stage 2 (i.e. cystic and sclerotic radiographic changes), three (6.1%) had stage 3 (i.e. subchondral lucency or crescent sign), 16 (32.7%) had stage 4 (flattening of the femoral head), 10 (20.4%) had stage 5 (i.e. joint space narrowing with or without acetabular involvement), and 16 (32.7%) had stage 6 (i.e. advanced degenerative changes).

**Figure 2:**
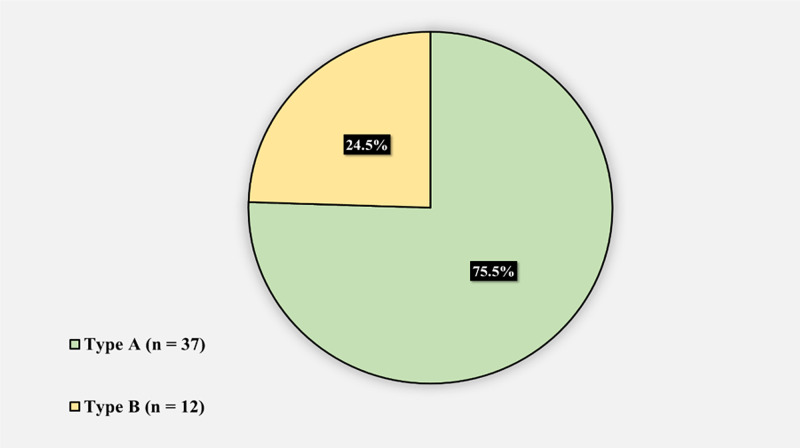
Radiological proximal femoral morphology using the Dorr classification

Figure 2 displays the radiological proximal femoral morphology using the Dorr classification of the SCD patients who underwent THA: 37 (75.5%) of the patients had type A morphology, and 12 (24.5%) had type B morphology. 

**Figure 3:**
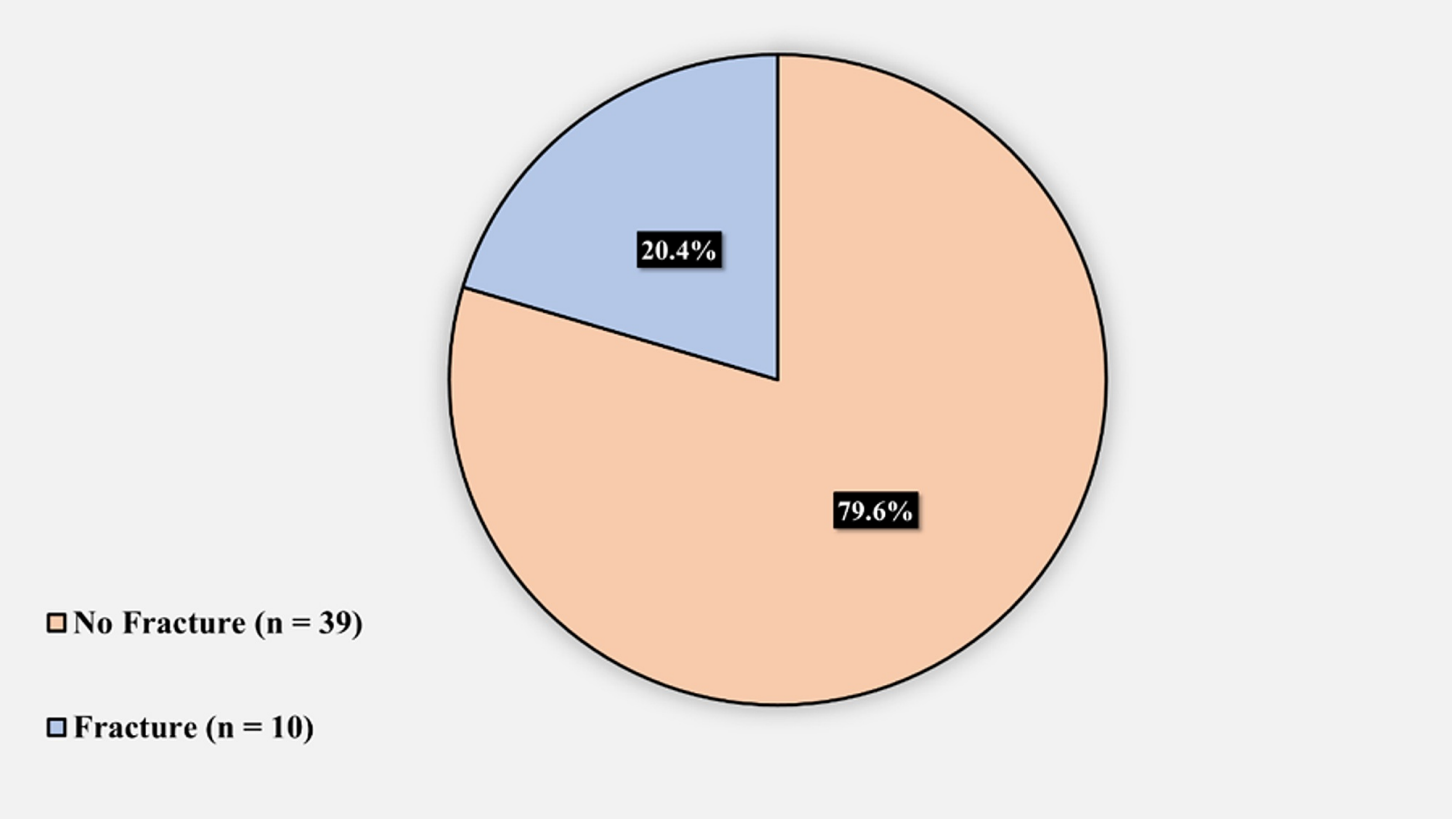
Prevalence of intra-operative periprosthetic femoral fractures in sickle cell disease patients who underwent total hip arthroplasty

Figure 3 demonstrates the prevalence of intra-operative periprosthetic femoral fractures in SCD patients who underwent THA: 10 (20.4%) had an intra-operative periprosthetic femoral fracture and 39 (79.6%) did not. 

**Figure 4:**
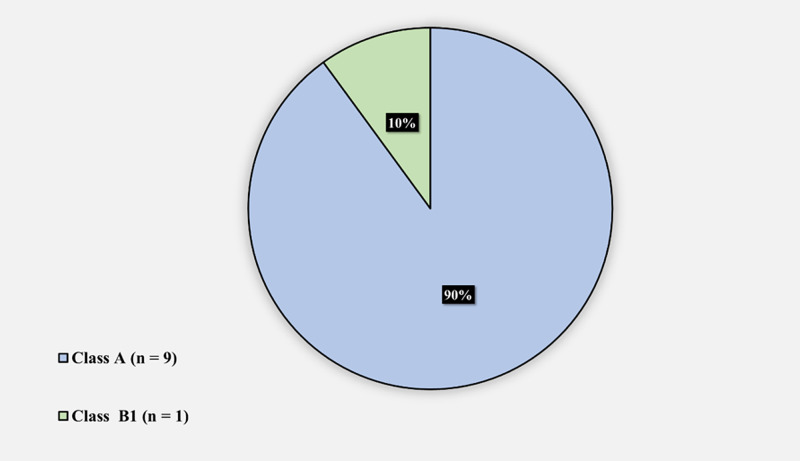
Periprosthetic femoral fracture according to the Vancouver classification system

Figure 4 demonstrates the periprosthetic femoral fracture Vancouver system classes in SCD patients who underwent THA: 9 (90%) had a class A femoral fracture and 1 (10%) had a class B1 femoral fracture. 

Table 3 represents the association between the incidence of intraoperative femoral fracture during THA in SCD patients and the demographic variables and operative profiles. There was a significant association between the operated site and the incidence of intraoperative femoral fracture (p = 0.035), and a higher rate of fracture was observed in patients operated on the right side compared to patients operated on the left side (35% vs. 10.3%). No significant association was found between the incidence of intraoperative femoral fracture during THA in SCD patients and the demographic variables or the other operative profile characteristics.

**Table 3:** Association between the incidence of intraoperative femoral fracture during total hip arthroplasty in sickle cell disease patients and the demographic variables and operative profiles

Demographic Characteristics		Incidence of Femoral Intraoperative Fracture During Total Hip Arthroplasty		
		No	Yes	P-Value
Gender				0.579
Male		12 (75%)	4 (25%)	
Female		27 (81.8%)	6 (18.2%)	
Age (mean, SD)		41.41 + 11.85	44.10 + 8.65	0.505
BMI				0.612
Underweight		3 (100%)	0 (0%)	
Normal BMI		21 (80.8%)	5 (19.2%)	
Overweight		9 (69.2%)	4 (30.8%)	
Class 1 obesity		6 (85.7%)	1 (14.3%)	
Perioperative Hemoglobin (mean, SD)		9.14 +2.08	8.54 + 1.76	
American Society of Anesthesiologist Class				0.237
1		3 (100%)	0 (0%)	
2		20 (71.4%)	8 (28.6%)	
3		16 (88.9%)	2 (11.1%)	
Operated Side				0.035*
Right		13 (65%)	7 (35%)	
Left		26 (89.7%)	3 (10.3%)	
Implant Type Used				0.609
Cemented		1 (100%)	0 (0%)	
Uncemented		38 (79.2%)	10 (20.8%)	
Avascular Necrosis Stage				0.837
2		3 (75%)	1 (25%)	
3		3 (100%)	0 (0%)	
4		13 (81.3%)	3 (18.8%)	
5		7 (70%)	3 (30%)	
6		13 (81.3%)	3 (18.8%)	

## Discussion

Patients with avascular necrosis related to SCD can be severely disabled by the status of their hip. Total hip arthroplasty remains the only surgical option for some of these patients given severe degenerative changes after collapse. Total hip arthroplasty for SCD patients can be a challenging procedure; they demonstrate high percentages of medical, intraoperative, and postoperative complications and implant failure. Because the need for THA following avascular necrosis in the Eastern Province of Saudi Arabia is high, the subsequent risk of periprosthetic fracture is common; therefore, it was crucial to conduct this study.

The study determined that 32.7% of the patients who underwent THA at King Fahad Hospital Hofuf had advanced degenerative changes due to avascular necrosis. Among these patients, 20.4% had intraoperative periprosthetic femoral fractures, 90% had class A femoral fractures, and 10% had class B1 femoral fractures. With our skilled SCD team and the multidisciplinary management of patients undergoing THA, our rates of intraoperative fracture are equal or close to those observed in the literature. However, our data did not indicate complete THA safety in this population. By comparing the outcomes of our patients with those reported in the literature, our results can help patients and orthopedic surgeons to better understand the relative risks and outcomes of this procedure in this population. However, even with adequate perioperative hydration, oxygen supply, warming, exclusion of infection sites, and conservative transfusion protocols to maintain appropriate Hb level, orthopedic surgeons must be prepared to deal with the high rate of intraoperative fracture.

The metaphyseal femoral morphology is typically distorted in SCD patients, characterized by thin trabeculae and cortices, medullary hyperplasia, low bone density, and patchy areas of bone sclerosis that can obliterate the femoral canal; therefore, the subsequent risk of periprosthetic fracture is greater [24]. The literature review determined that Dorr type C has a higher risk of fracture. In our study, 37 (75.5%) patients had type A, 12 (24.5%) had type B. We recommend further studies to investigate this aspect. For example, Kenanidis et al. [24] recommended facilitating femoral reaming by introducing a 4.5-mm drill bit or a high-speed burr under an image intensifier and preparing the femoral bone to accommodate flexible intramedullary guide wires to perform cannulate intramedullary reaming.

Intraoperative periprosthetic fractures are becoming more common given the increased prevalence of revision THA and the use of uncemented fixation [25]. Many other studies have also demonstrated that uncemented implants are more associated with intraoperative complications, and almost all reported intraoperative femoral fractures were associated with uncemented THA [26]. In our study, 48 patients had an uncemented implant, and only one had cemented. Different implant options, such as short or long stems, revision options, and plates for periprosthetic fractures must be available to deal with intraoperative complications.

We found a significant association between the operated site and the incidence of intraoperative femoral fracture: a higher rate of fracture was observed in patients operated on the right side compared to the left side. Maybe, this can be explained as all of our surgeons and assistants are right-handed, so they will have better control when operating on the left side as compared to the right side. As far as we know, this is the first study to find this, so we recommend further studies to investigate this aspect. However, by comparing the gender, age, BMI, and ASA classification of our patients with those reported in the literature, we found no significant association between the incidence of intraoperative femoral fracture during THA in SCD patients.

The main limitation of this study was the low number of participants due to the small population group compared to the general population; therefore, we recommend further multicenter studies to be conducted in Saudi Arabia's, Eastern Province. This was a retrospective study, so the analyses of the surgical complications were limited by a lack of clinical detail. Additionally, the published studies we reviewed contained low levels of evidence: the majority were non-randomized retrospective case series reporting outcomes over the last 40-50 years [26-30].

## Conclusions

The prevalence of periprosthetic intraoperative fracture among SCD patients at King Fahad Hospital Hofuf was 20.4% during the study period, January 2015 to September 2020. Even with adequate perioperative management, orthopedic surgeons must be prepared to deal with the high rate of intraoperative fracture. Of the patients, 90% had a class A femoral fracture, and 10% had a class B1 femoral fracture. No significant association was found between the incidence of intraoperative femoral fracture in SCD patients and the demographic variables or the operative profile characteristics. However, a significantly higher rate of fracture was observed in patients operated on the right side compared to patients operated on the left side. Finally, we recommend a future multicenter study to be conducted in Saudi Arabia's, Eastern Province.
